# Pseudotumor of the Maxillary Sinus in a Child with Von Willebrand Disease

**DOI:** 10.22038/IJORL.2022.58284.3004

**Published:** 2022-11

**Authors:** Martha Lucía Gutiérrez Pérez, Juan Antonio Lugo Machado, Natalia Barreto Niño, Luis Alfonso Paredes Bastos, David Fernando Acevedo Acevedo

**Affiliations:** 1 *Medical Doctor. Universidad El Bosque, Colombian School of Medicine, Otorhinolaryngology Interest Group UEB (ORLIG-UEB), Bogotá, Colombia.*; 2 *Departement of Otorhinolaryngology Head and Neck Surgery, Hospital General de Obregón, Sonora, Secretaria de Salubridad y Asistencia, Ciudad Obregón, Sonora, México.*

**Keywords:** Child, Hemophilic pseudotumor, Mandibular diseases, Pediatrics, Von Willebrand diseases.

## Abstract

**Introduction:**

Mandibular pseudotumors, also known as blood cysts, are rare complications which occur more frequently in patients with an associated bleeding disorder such as hemophilia.

**Case Report:**

We present a case of a 2-year and 6-month-old patient with a hemophilic pseudotumor associated with Von Willebrand's disease, who consulted the emergency room due to spontaneous increase in volume of the left maxillary region, with no previous relevant medical history.

**Conclusions:**

Different imaging studies were carried out to characterize the lesion, providing the necessary information for the correct approach. Due to the low prevalence of this complication, we believe it is of vital importance to understand the adequate management in this patient population.

## Introduction

Hemophilic pseudotumor is caused by a hemorrhagic diathesis that manifests itself in bone and soft tissues, and is considered to be an infrequent complication that can become serious (1). 

The pseudotumor is described as being similar to a progressive cystic tumor, which involves the extra-articular musculoskeletal system, causing pressure on the adjacent tissues, which can lead to repercussions in the skin and muscle vasculature (2,3). 

It is estimated that this complication occurs in 1% to 2% of patients with a history of severe hemophilia, and a lower percentage in those with Von Willebrand disease, thus making this a very rare case (1,2).

It is important to understand that Von Willebrand's disease is an autosomal dominant congenital bleeding disorder, consisting of a quantitative and qualitative deficiency of factor VIII/Von Willebrand factor complex, and inefficient plasma coagulability leading to impaired hemostasis (1).

The objective of this article is to present an infrequent clinical case about a female child with a history of Von Willebrand disease who presented with a complication of maxillary pseudotumor, and highlight the adequate diagnosis and management of these cases.

## Case Report

Prior written informed consent was obtained from the mother of the patient who participated in this case. We present a 2-year-6-month-old female patient, who arrived to the pediatric emergency room due to spontaneous increase in volume of the left maxillary region and the left gingivo-labial region, with increased volume of the canine space, without changes in skin color and integuments, nor signs of hyperthermia. The patient’s clinical history was remarkable for smoking by her biological father (10 cigarettes per month), and no other significant family history or pregnancy complicationswere noted. 

**Fig 1:**
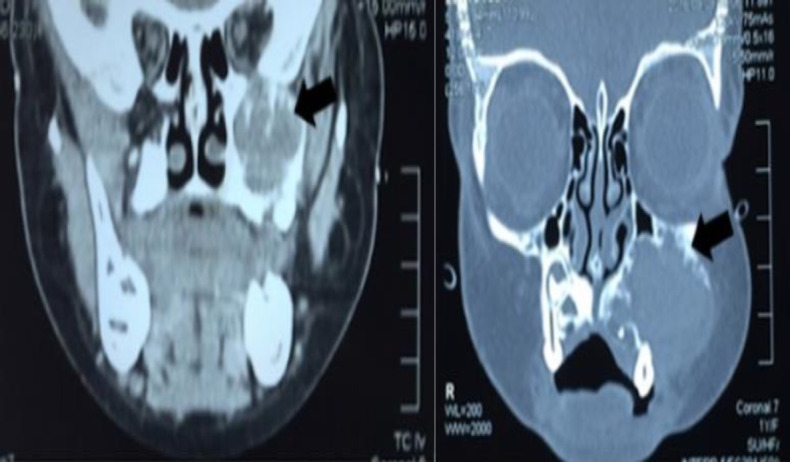
CT of the nose and paranasal sinuses. Shows findings of an expansive lesion in the maxillary sinus cavity that erodes and causes lysis of the maxillary sinus walls

The computed tomography (CT) (Figure 1) of the nose and paranasal sinuses showed an expansive lesion in the maxillary sinus cavity that eroded and caused bone lysis of the left maxillary sinus walls, with no signs of contrast material uptake. 

A magnetic resonance arteriography ruled out a tumor of vascular origin, and a hypointense lesion located in the maxillary antrum was also observed.

**Fig 2:**
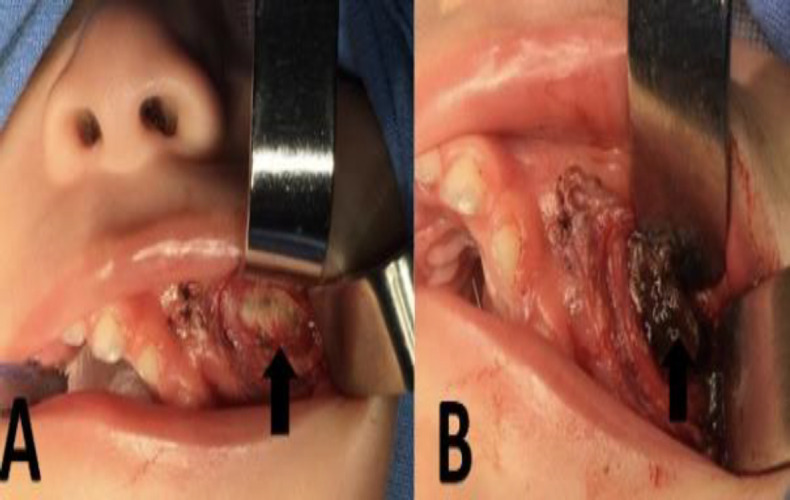
Biopsy. Submucosal plane with increased volume in the left gingivo-labial region (surgical image A). Large collection of coagulated blood (surgical image B)

Laboratory tests showed prothrombin time of 12.1 seconds, partial thromboplastin time of 34.0 seconds, as well as a normal platelet count. A biopsy was done of clot fragments removed from the left maxillary sinus cavity and also of the tumor (Figure 2) due to the possible differential diagnosis of an expansive malignant process, which revealed irregular tissue fragments named as “left maxillary tumor” of 3 x 2.5 x 0.5 centimeters, covered by areas of fibroblast proliferation and dense connective tissue similar to a scar. Histopathologically, areas of proliferating fibroblasts and dense fibrous connective tissue were found. 

24 hours after surgery, an increase in volume was noted due to hematoma in the surgical area that improved 72 hours after the application of plasmapheresis. Due to the improvement seen after the plasmapheresis, the possibility of a deficiency of a coagulation factor was taken into account, therefore hematology was consulted and the patient was found to have low factor VIII and decreased Von Willebrand's factor, confirming the suspicion of Von Willebrand's disease. Therefore, plasma transfusions were given in order to correct the deficiency of this coagulation factor.

## Discussion

Pseudotumors arise as a complication of bleeding disorders, and consist of a collection of encapsulated blood generated after recurrent bleeding in bones or soft tissues (2), and can be present in different stages of organization, always surrounded by a thick fibrous capsule (4). Von Willebrand’s disease is caused by Von Willebrand factor deficiency, which is a clotting factor that mediates platelet adhesion to sites of vascular injury and is in charge of binding and stabilizing the procoagulant protein factor VIII (5). 

This interaction between platelets and the vessel wall alters primary hemostasis, leading to heavy and continuous bleeding after any type of injury (5,6). Pseudotumors have been reported to be caused by prolonged bleeding after minor injuries and by surgical procedures (7), especially in patients with coagulopathies. Therefore, one should assure that the patient’s clinical history is thoroughly reviewed for evidence of bleeding disorders (5).

The increased pressure generated by the hematoma leads to a weathering of the contiguous tissues, having repercussions on the neighboring skeletal muscle and vascular structures. This occurs mostly in adult long bones, and in the iliac, vastus lateralis and soleus muscles (4). Less common sites of involvement include the orbital region, mandible, clavicle, skull, spinal canal, and small bones of the hand (2,3).

The reported cases of hemophilic pseudotumor of the upper and lower jaw have occurred in pediatric patients (with an age range of 6 months to 34 years, mean age 11.1 years) (7), with the most frequent site of presentation being the mandible (4). The reasons for pseudotumors arising in the jaw have not been recorded so far (5).

The classic clinical presentation is painless swelling unless it involves nerve structures, pathologic fractures, or aggregated infections (8). When the involvement is extensive, ischemia with subsequent bone destruction can ensue (5). Adjacent necrosis, functional disability, erosions in different tissues, and compartment syndrome are complications that can be avoided if a timely diagnosis is made with the help of radiological evaluations (8).

They usually present as very well defined unilocular or multilocular radiolucent lesions, with thin septa or trabeculations. There may be intralesional foci of dystrophic calcifications, cortical thickening or thinning, and marginal sclerosis (7).The CT scan can show areas of loculation, a range of bone destruction, and the displacement of neurovascular structures. Similarly, magnetic resonance imaging (MRI) can be utilized to evaluate small neurovascular structures (4). The MRI findings are characterized by reviewing both hyper and hypointense areas in T1 and T2 sequences, suggesting hematic content corresponding to the blood clot, surrounded by hypointense fibrous tissue. Conducting a study of vascular structures such as CT angiography is useful to determine that the tumor is avascular and helps arrive at the diagnosis of pseudotumor (8).

**Fig 3:**
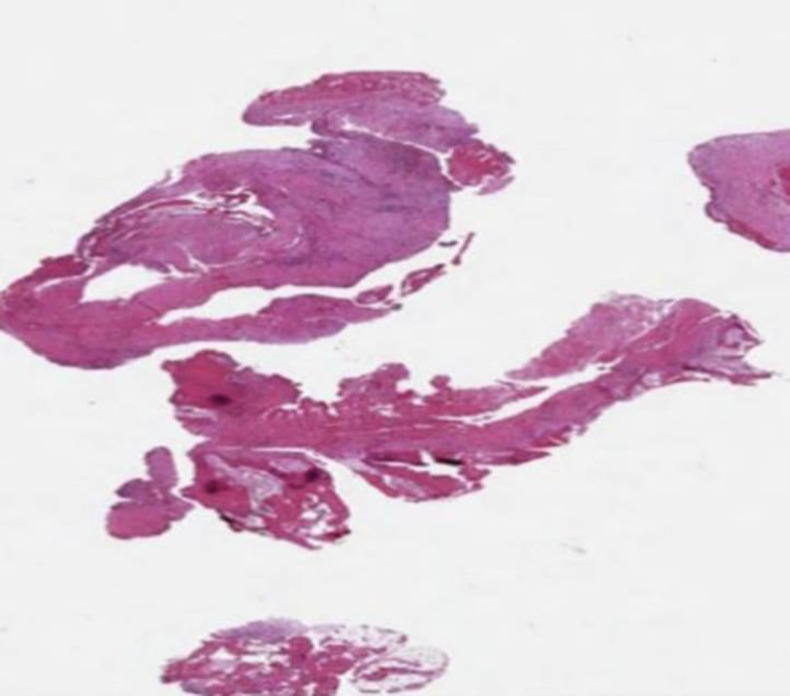
Low-power photomicrograph. It reveals abundant hemorrhagic material and cystic areas, fibroblast lining, and scar-like connective tissue with hematoxylin-eosin staining

**Fig 4:**
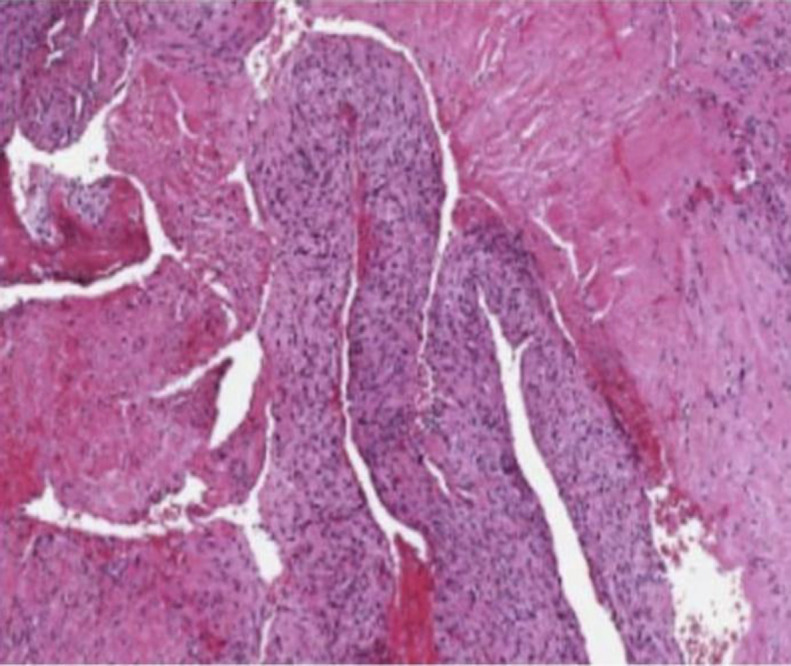
Medium power photomicrograph. Shows areas of fibroblast proliferation and dense connective tissue with hematoxylin-eosin staining

Histological examination of hemophilic pseudotumors (Fig 3 and 4) can show organized fibrous tissue, blood clots, fragments of cancellous bone, neovascularization, osteoid neoformation, and accumulations of inflammatory cells (predominantly histiocytic, with considerable amounts of hemosiderin) (4,5).

As mentioned previously, trauma is an etiological factor to take into account, however, cases have been reported in which there is an absence of any history of trauma, leading to a possible suspicion of dental eruption to be a cause of the bleeding (5).

For the treatment of hemophilic pseudotumor, it is important to carry out proper prevention in those patients with bleeding disorders, which must be accompanied by imaging tests, together with the correct management of the underlying pathology. In our case of Von Willebrand's disease, substituting the missing factors would be the correct intervention of choice (8,9).

Most researchers have advised for a cautious approach to the treatment of pseudotumors of the mandible. In cases when a conservative treatment has failed, cryotherapy, aminocaproic acid injection, or ethanol embolization, as well as surgical intervention, are required (10). Surgery is an option for the treatment of hemophilic pseudotumors, which is usually carried out in a specialized center for the management of coagulative disorders. 

The approach to this complication can be hemimandibular resection, enucleation, curettage with or without tooth extractions, aspiration, and replacement therapy (4,8). Regarding the differential diagnosis, adenocystic carcinoma, squamous cell carcinoma, ameloblastoma, esthesioneuro- blastoma and lymphoma should be taken into account. For this, contrast enhancement should be observed as most will cause bone destruction rather than expansion (2).

## Conclusion

Considering the infrequency of this complication, it is important to evaluate the pseudotumor as a possible differential diagnosis in lesions with mass effect in the context of patients with blood dyscrasias. It is important to emphasize the use of imaging studies for diagnosis, with characterization of the lesion and the choice of an appropriate treatment, without neglecting the importance of laboratory studies to recognize if there is a history of hemorrhagic diseases.
